# Attentional Bias to Facial Expressions of Different Emotions – A Cross-Cultural Comparison of ≠Akhoe Hai||om and German Children and Adolescents

**DOI:** 10.3389/fpsyg.2020.00795

**Published:** 2020-04-28

**Authors:** Cordelia Mühlenbeck, Carla Pritsch, Isabell Wartenburger, Silke Telkemeyer, Katja Liebal

**Affiliations:** ^1^Department of Psychology, Brandenburg Medical School Theodor Fontane, Neuruppin, Germany; ^2^Comparative Developmental Psychology, Department of Education and Psychology, Freie Universität Berlin, Berlin, Germany; ^3^Graduate School “Languages of Emotion”, Freie Universität Berlin, Berlin, Germany; ^4^Department of Linguistics, Cognitive Sciences, University of Potsdam, Potsdam, Germany

**Keywords:** attentional bias, fear bias, emotions, facial expressions, cross-cultural comparison, ≠Akhoe Hai||om, Germans, adolescents

## Abstract

The attentional bias to negative information enables humans to quickly identify and to respond appropriately to potentially threatening situations. Because of its adaptive function, the enhanced sensitivity to negative information is expected to represent a universal trait, shared by all humans regardless of their cultural background. However, existing research focuses almost exclusively on humans from Western industrialized societies, who are not representative for the human species. Therefore, we compare humans from two distinct cultural contexts: adolescents and children from Germany, a Western industrialized society, and from the ≠Akhoe Hai||om, semi-nomadic hunter-gatherers in Namibia. We predicted that both groups show an attentional bias toward negative facial expressions as compared to neutral or positive faces. We used eye-tracking to measure their fixation duration on facial expressions depicting different emotions, including negative (fear, anger), positive (happy), and neutral faces. Both Germans and the ≠Akhoe Hai||om gazed longer at fearful faces, but shorter on angry faces, challenging the notion of a general bias toward negative emotions. For happy faces, fixation durations varied between the two groups, suggesting more flexibility in the response to positive emotions. Our findings emphasize the need for placing research on emotion perception into an evolutionary, cross-cultural comparative framework that considers the adaptive significance of specific emotions, rather than differentiating between positive and negative information, and enables systematic comparisons across participants from diverse cultural backgrounds.

## Introduction

A large body of research suggests that humans pay more attention to negative than positive information (e.g., Baumeister et al., 2001; Rozin and Royzman, 2001). The enhanced sensitivity to negative information – resulting in increased alertness and the mobilization of attentional resources – is most likely an evolutionary adaptive behavior, as the ability to successfully detect and appropriately respond to threatening and potentially harmful situations increases the probability of survival (Öhman and Mineka, 2001). Positive information, on the other hand, promotes flexible and explorative behavior, and as a result, supports social bonding and positive interactions (Fredrickson, 1998). Unlike in case of neglecting negative information, the consequences of a missed opportunity to react appropriately to positive information seem much less severe (Baumeister et al., 2001).

Given the evolutionary significance of the fast detection of and appropriate reaction to potential threats (Öhman and Mineka, 2001), it seems likely that the attentional bias to negative emotions is a universal trait shared by all humans regardless of their cultural background. However, researchers have largely focused on humans from industrialized, Western societies, who are not representative for the human species (Henrich et al., 2010). Although some studies showed that emotional facial expressions are not universally recognized (Jack et al., 2012; Gendron et al., 2014; Crivelli et al., 2016) and that humans attend to positive or negative information differently depending on their cultural backgrounds (Grossmann et al., 2012), such cross-cultural comparisons including several human populations from diverse cultural, social, and ecological backgrounds remain scarce.

Furthermore, the notion of an exclusive, biologically prepared bias toward negative information has been challenged, as meta-analyses also confirmed a bias toward positive information (Pool et al., 2016), or demonstrated that the negativity bias is influenced by, for example, anxious concerns, type of negative information, or age (Bar-Haim et al., 2007; van Rooijen et al., 2017; Lisk et al., 2019). Thus, to better understand these apparently inconsistent findings, it is crucial to understand developmental pathways of attentional biases. However, unlike for adults, there is substantially less research with children and adolescents (Vaish et al., 2008). The bias toward negative information emerges early in ontogeny (Leppänen and Nelson, 2012), since after an initial positivity bias (Vaish et al., 2008), infants between 5 and 7 months of age pay more attention to negative information, such as fearful faces (Grossmann and Jessen, 2017). From about 4 years of age, children seem to prefer both negative and positive emotional stimuli over neutral information (Elam et al., 2010; Burris et al., 2017), while adolescents show a bias toward negative emotions (Grose-Fifer et al., 2013). In adulthood, there is substantial evidence for a bias toward negative information across different domains, such as social interactions and relationships, learning or emotion processing (Baumeister et al., 2001). Several studies show, however, that a negativity bias in children, adolescents and adults is specifically found in more anxious individuals (Bar-Haim et al., 2007; Shechner et al., 2013). In older adults, there is a shift toward a bias for positive information, although this seems to differ across cultural contexts (Fung et al., 2008). Taken together, at different times in ontogeny, humans preferentially pay attention to negative and/or positive information, with the overall pattern that – at least in Western societies – the bias toward negative emotions emerges in the second half of infants’ first year of life, and, although also positive information is preferred over neutral stimuli (Pool et al., 2016), a pronounced positive bias only emerges again in older adults.

To investigate attentional biases, researchers often focus on emotional information and use facial expressions of basic emotions to compare humans’ responses to negative, positive, and neutral faces. Unlike the proposed general bias to negative information (Rozin and Royzman, 2001), there is increasing evidence that humans’ responses to faces vary across types of negative emotions (for reviews, see Frischen et al., 2008; Vaish et al., 2008; Yiend, 2010). However, studies vary in their conclusions with regard to which negative emotion attracts most attention. For example, while Williams et al. (2005) found that angry faces are detected faster than fearful faces, many studies report a fear bias (Vaish et al., 2008), supported by neurobiological evidence showing a stronger activation of the amygdala in response to fearful as compared to angry faces (Whalen et al., 2001). Interestingly, some studies suggest that threat-related stimuli, such as angry faces, are even avoided by children and adolescents, particularly by anxious youths (Lisk et al., 2019). To compare findings across studies and to identify general patterns, however, is difficult, as most studies focus on one type of negative emotion (either anger or fear) and therefore do not investigate if responses differ depending on the type of negative emotion (e.g., Hansen and Hansen, 1988; Rossignol et al., 2013).

We aimed at filling two of these several gaps in research on attentional biases: the lack of research with humans from non-Western societies, and the limited knowledge about the processing of different types of negative emotion in children and adolescents. We studied two very distinct cultural groups: the ≠Akhoe Hai||om, who are semi-nomadic egalitarian hunter-gatherers in northern Namibia; and Germans from two large cities as representatives of a Western industrialized society. We used eye-tracking in a free viewing task to investigate if the groups’ gazing patterns on facial expressions varied depending on the type of the depicted emotion, and compared two negative emotions (fear, anger) with a positive emotion (happy) and neutral faces. We hypothesized that given the evolutionary significance of negative, potentially threatening information, participants from both cultural backgrounds should look longer at negative facial expressions compared to neutral or positive faces. Although we cannot claim to identify universal behaviors based on two samples, we suggest that if we find an attentional bias toward negative information in both groups, which differ with regard to several factors, such as dwelling, subsistence, and social organization, it seems at least likely that this trait is shared by many humans regardless of their cultural backgrounds.

## Materials and Methods

### Participants

We focused on adolescents and children as we were not able to recruit sufficient numbers of adults from the ≠Akhoe Hai||om. Using opportunity sampling, we first collected the data in Namibia and tested adolescents and children who were available and willing to participate. It is important to note that determining the exact ages of the ≠Akhoe Hai||om was difficult, since many children are not officially registered when they are born, and therefore exact dates of birth days are often not known. Second, we tested adolescents and children in Germany and tried to match participants in terms of age and gender to the ≠Akhoe Hai||om sample.

The ≠Akhoe Hai||om in northern Namibia are semi-nomadic hunter-gatherers, characterized by egalitarian social structures and the common practice of sharing of resources (Widlok, 1999). Their traditional lifestyle is changing, as they have become increasingly sedentary and have taken up alternative subsistence strategies, like gardening or animal husbandry, and formalized schooling has been introduced. Participants were recruited from the Khomxa Khoeda Primary School at Farm 6 and comprised 30 participants (15 males, 15 females; mean age = 11.93 years, *SD* = 2.87, age range 7–19 years).

Germany is an industrialized Western European nation that values individual independence. The German sample consisted of 21 participants (9 males, 12 females, mean age = 13.76 years, *SD* = 1.48, age range 12–18 years), who were recruited from two schools in two large cities (Marion-Dönhoff-Gymnasium, Hamburg; Bertolt-Brecht High School, Berlin).

Although it has been shown that affectivity or depressed mood may affect attention for positive facial expressions (e.g., Isaac et al., 2014), we were not able to assess this in our participants, as there are no standardized, culture-fair questionnaires for measuring affectivity in the ≠Akhoe Hai||om.

The study was conducted in accordance with the Declaration of Helsinki and the ethical guidelines of the German Psychological Society. The study did not require approval by an Institutional Review Board, as it did not involve any invasive techniques, ethically problematic procedures, or deception [see the regulations on freedom of research in the German Constitution, §5 (3)]. Permission to conduct this study with the ≠Akhoe Hai||om was obtained from the “Working Group of Indigenous Minorities in Southern Africa” (WIMSA) in Windhoek, and the local school’s principal, Efraim Kavetuna. Prior to testing, each participant was informed about the background and procedure of the study by a video recording in their native language, and gave their informed consent verbally. In Germany, parents gave their written informed consent.

### Stimuli

We selected pictures (440 × 550 pixels) of 14 German adults (7 females) showing negative (fearful, angry), positive (happy), and neutral faces from the FACES database (Ebner et al., 2010)^1^. Thus, unlike German participants, the ≠Akhoe Hai||om looked at stimuli from a different ethnical group. Although there are databases with stimuli representing a greater ethnical diversity (e.g., NimStim; Tottenham et al., 2009), there is none with facial expressions of the ≠Akhoe Hai||om. Therefore, we used stimuli from the FACES database, since it depicts naturalistic facial expressions of amateur actors rather than professionals, and the photographs, standardized in size, color and background, have been extensively rated with regard to the type of depicted emotional expression (Ebner et al., 2010).

To enable the comparison of fixations between two emotions, each stimulus consisted of a pair of pictures, with each picture showing a different facial expression of the same person (e.g., person A with a happy face and person A with a fearful face). The picture pairs were presented on gray background, separated by a gap of 10 pixels. Each facial expression was combined with every other facial expression, resulting in six possible picture combinations (fear-angry, fear-happy, fear-neutral, angry-happy, angry-neutral, happy-neutral). The position of each facial expression (left or right) was counterbalanced and the order of presenting these picture pairs was randomized. Each participant saw an equal number of pictures of each facial expression and their combinations with the corresponding other facial expressions.

### Procedure

All participants were tested in a separate room in their schools. A table-mounted, monitor-integrated eye-tracker (Tobii T60) was used to measure the participants’ fixations with an infrared corneal reflection system. Participants were seated in front of the monitor (distance: 60–70 cm), on which the stimuli were presented. As we used a free-viewing task, the participants were only informed that a series of facial expressions will be shown on a screen we asked them to watch (Germans: verbal information; ≠Akhoe Hai||om: video-recorded information in their native language). Before the experiment started, we conducted an automated five-point calibration (with a moving red dot on white background) and only started testing after successful calibration. To start the experiment, the participant’s gaze was centered on the screen by presenting a fixation cross for 500 ms. Then a picture pair appeared for 3,000 ms, followed by a fixation cross shown for 500 ms to again center the participant’s gaze, followed by the next picture pair. In total, 168 different trials were presented (28 trials per condition of each picture pair) to each participant in one session, which lasted ~15–20 min.

### Data Analysis

Raw data were extracted (see Supplementary Data Sheet 1) and then collapsed for analysis using the software Matlab^®^ (R2014b). For the analysis, we defined one of the two facial expressions as the target emotion and the other as the distractor emotion. Depending on the focus of the analysis, each emotion was considered either as target or distractor. We defined each of the two facial expressions in a trial as a separate area of interest (AoI), and measured the fixation duration separately for each of the two AoIs. A fixation was scored if the gaze remained stationary within a radius of 50 pixels for at least 100 ms. The measurement of first fixations was not suitable, as this would have required a more controlled testing situation (e.g., restriction of head movements, control of pre-trial fixation position, and no distractions, such as noise), which was not possible to achieve. Therefore, we focused on fixation duration over the course of stimulus presentation, which enabled us to derive maintained attention but also avoidance over the time of stimulus presentation (Lisk et al., 2019).

We conducted two sets of analyses: first, we compared the average cumulative fixation duration on each target emotion in comparison to the average cumulative fixation duration on all corresponding distractors combined (e.g., target = happy vs. all distractors = neutral + fear + anger). Second, we compared the mean fixation duration on each target emotion with the mean fixation duration on a specific distractor (e.g., target = happy vs. distractor = anger). While we conducted the first analysis to control for the possibility that the fixation duration on a specific target emotion was influenced by the simultaneously presented distractor emotion, the second analysis enabled us to directly compare fixation durations between two different emotions. Because mean ages varied between our samples, we first tested if age had an influence on fixation duration. We found no effect and therefore excluded this variable from further analyses.

To test if fixation durations differed across emotions, we compared a particular emotion combination consisting of the target emotion and distractor(s) by fitting a linear mixed model in R (3.1.1, R Core Team, 2013) using the function lmer of the R-package lme4 (Baayen, 2008; Bates et al., 2014). We ran a linear mixed model with no intercept and no slopes, with fixation duration as dependent variable, and the different levels of the predictor emotion combination as fixed effects, as well as subject as random effect to account for individual differences. Since the levels of the variable emotion combination were not independent, we allowed for all possible correlations. *P*-values were derived by comparing the full model with all levels of emotion combination included to a reduced model with the specific combination excluded. The significance of this comparison was tested using a likelihood ratio test (R function anova with argument test set to “Chisq”).

## Results

### First Analysis: Target Emotion vs. All Distractors Combined

**FIGURE 1:**
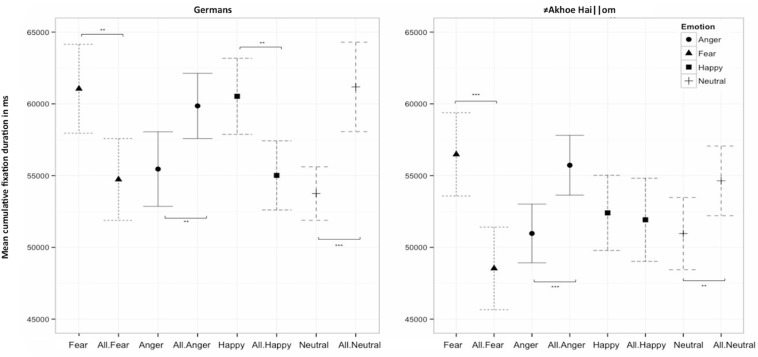
Mean cumulative fixation duration for the comparison of the target emotion (e.g., Fear) vs. all distractors combined (e.g., All.Fear) for Germans and ≠Akhoe Hai||om. Scales show the mean fixation duration in milliseconds. Significant differences are indicated by asterisks (**p < 0.01, ***p < 0.001).

Germans fixated on both fearful and happy faces significantly longer than on all other corresponding distractors combined (fear vs. distractors: χ^2^ = 5.37, df = 1, *p* = 0.021; happy vs. distractors: χ^2^ = 6.54, df = 1, *p* = 0.011), while they fixated on neutral and angry faces significantly shorter compared to their respective distractors (neutral vs. distractors: χ^2^ = 10.97, df = 1, *p* = 0.001; anger vs. distractors: χ^2^ = 5.63, df = 1, *p* = 0.018) (Figure 1 and Table 1). Similarly, the ≠Akhoe Hai||om fixated on fearful faces longer than on their distractors (fear vs. distractors: χ^2^ = 13.30, df = 1, *p* < 0.001), while they fixated on both neutral and angry faces significantly shorter than on their distractors (neutral vs. distractors: χ^2^ = 5.38, df = 1, *p* = 0.020; anger vs. distractors: χ^2^ = 15.76, df = 1, *p* < 0.001). While both groups exhibited similar fixation patterns for fearful, angry, and neutral faces, they responded differently to happy faces, since unlike German participants, the ≠Akhoe Hai||om did not fixate on them longer compared to all other emotions (happy vs. distractors:χ^2^ = 0.11, df = 1, *p* = 0.738).

**TABLE 1:** Estimates for the mean cumulative fixation duration (in ms) on the target emotion vs. all distractors (see also Figure 1) calculated for both groups.

	**Germans**					**≠Akhoe Hai||om**				
**Emotion combination**	**Estimate**	**Std. Error**	**Upper CI**	**Lower CI**	***t*-value**	**Estimate**	**Std. Error**	**Upper CI**	**Lower CI**	***t*-value**
Fear	61053	1581	64151	57956	38.6	56482	1480	59382	53581	38.2
Anger	55458	1325	58056	52861	41.8	50970	1043	53015	48925	48.9
Happy	60529	1353	63181	57877	44.7	52406	1337	55027	49785	39.2
Neutral	53750	951	55614	51886	56.5	50960	1285	53479	48442	39.7
All.Fear	**54734**	**1454**	**57585**	**51884**	**37.6**	**48534**	**1470**	**51416**	**45652**	**33.0**
All.Anger	**59857**	**1160**	**62130**	**57584**	**51.6**	**55725**	**1063**	**57809**	**53641**	**52.4**
All.Happy	**55016**	**1228**	**57424**	**52608**	**44.8**	51923	1477	54818	49027	35.2
All.Neutral	**61183**	**1591**	**64301**	**58066**	**38.5**	**54636**	**1237**	**57061**	**52211**	**44.2**

*Values are derived from the linear mixed model, predicting estimates, standard errors (Std. Error), upper and lower confidence intervals (Upper CI, Lower CI), and t-values for the mean cumulative fixation duration on a specific target emotion (Fear, Anger, Happy, Neutral) compared to all other distractor emotions (All.fear, fear vs. neutral, happy, anger; All.anger, anger vs. neutral, happy, fear; All.happy, happy vs. neutral, fear, anger; All.neutral, neutral vs. fear, anger, happy). Significant comparisons are highlighted in bold letters. Models were calculated separately for Germans and ≠Akhoe Hai||om, respectively.*

### Second Analysis: Target Emotion vs. Specific Distractor

**FIGURE 2:**
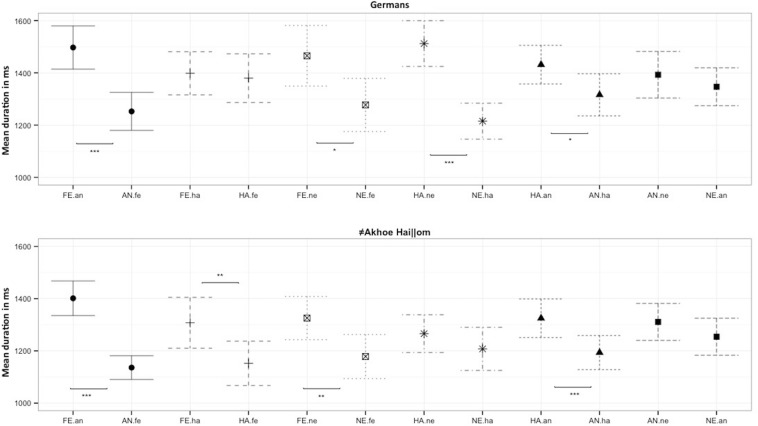
Mean fixation duration for the comparison of a target emotion (in capital letters) vs. a distractor emotion (in lower case letters) for Germans and the ≠Akhoe Hai||om. fe, fear; an, anger; ha, happy; ne, neutral. Scales show the mean fixation duration in milliseconds. Significant differences are indicated by asterisks (*p < 0.05, **p < 0.01, ***p < 0.001).

Both populations fixated on fearful expressions longer than on angry and neutral faces (≠Akhoe Hai||om: fear vs. anger χ^2^ = 31.49, df = 1, *p* < 0.001; fear vs. neutral χ^2^ = 4.98, df = 1, *p* = 0.026; Germans: fear vs. anger χ^2^ = 13.69, df = 1, *p* < 0.001; fear vs. neutral χ^2^ = 3.35, df = 1, *p* = 0.067), and on angry faces shorter than happy faces, although this was only a trend for Germans (≠Akhoe Hai||om: χ^2^ = 7.99, df = 1, *p* = 0.005; Germans: χ^2^ = 3.36, df = 1, *p* = 0.067). Unlike Germans, the ≠Akhoe Hai||om fixated on fearful faces significantly longer compared to happy faces (≠Akhoe Hai||om: χ^2^ = 6.28, df = 1, *p* = 0.012, Germans: χ^2^ = 0.10, df = 1, *p* = 0.740). Unlike the ≠Akhoe Hai||om, Germans fixated on happy faces longer than on neutral faces (Germans: χ^2^ = 17.84, df = 1, *p* < 0.001, ≠Akhoe Hai||om: χ^2^ = 0.14, df = 1, *p* = 0.723). In sum, resembling the pattern found in the first analysis, both groups fixated on fearful faces longer than on angry or neutral faces, and on angry faces shorter than on happy faces (Figure 2 and Table 2), while they varied to some extent in their responses to happy faces.

**TABLE 2:** Estimates for the mean fixation duration (in ms) on a target emotion vs. a specific distractor emotion (see also Figure 2) for both groups.

	**Germans**					**≠Akhoe Hai||om**				
**Emotion combination**	**Estimate**	**Std. Error**	**Upper CI**	**Lower CI**	***t*-value**	**Estimate**	**Std. Error**	**Upper CI**	**Lower CI**	***t-*value**
Fear.an	**1497**	**42.2**	**1580**	**1414**	**35.5**	**1401**	**33.8**	**1468**	**1335**	**41.4**
Anger.fe	**1253**	**37.1**	**1325**	**1180**	**33.8**	**1136**	**23.3**	**1182**	**1091**	**48.9**
Fear.ha	1399	42.2	1481	1316	33.2	**1308**	**49.7**	**1405**	**2110**	**26.3**
Happy.fe	1380	47.5	1473	1287	29.0	**1152**	**43.3**	**1237**	**1068**	**26.6**
Fear.ne	**1465**	**59.2**	**1581**	**1349**	**24.8**	**1326**	**42.2**	**1408**	**1243**	**31.4**
Neutral.fe	**1277**	**51.9**	**1379**	**1176**	**24.6**	**1178**	**43.0**	**1263**	**1094**	**27.4**
Happy.ne	**1512**	**44.6**	**1600**	**1425**	**33.9**	1266	36.9	1338	1194	34.3
Neutral.ha	**1215**	**35.2**	**1284**	**1146**	**34.6**	1208	42.1	1290	1125	28.7
Happy.an	**1437**	**37.8**	**1506**	**1358**	**37.9**	**1325**	**37.7**	**1399**	**1251**	**35.2**
Anger.ha	**1316**	**41.1**	**1397**	**1235**	**32.0**	**1194**	**33.3**	**1259**	**1128**	**35.8**
Anger.ne	1393	45.5	1482	1304	30.6	1311	36.1	1382	1240	36.3
Neutral.an	1346	37.0	1419	1274	36.4	1254	36.2	1325	1183	34.7

*Values are derived from linear mixed models, predicting estimates, standard errors (Std. Error), upper and lower confidence intervals (Upper CI, Lower CI), and t-values of the mean fixation duration on a particular target emotion (Fear, Anger, Happy, Neutral) compared to another specific distractor emotion (-.fe, -.an, -.ha, -.ne). Targets begin with capital letters, followed by distractors abbreviated in lower case letters (fe, fear; an, anger; ha, happy; ne, neutral). Significant comparisons are highlighted in bold letters. Models were calculated separately for Germans and ≠Akhoe Hai||om, respectively.*

## Discussion

We expected that participants from both cultural backgrounds would look longer at negative facial expressions compared to neutral or positive faces. In line with this expectation, both the ≠Akhoe Hai||om and Germans fixated on fearful expressions longer, regardless of the emotion they were combined with. Contrary to our prediction, both groups fixated on angry faces significantly shorter than on all other emotions. Thus, our hypothesis was only partially confirmed, since we did not find a general attentional bias toward negative emotions, as participants of both groups attended more to fearful than angry facial expressions.

To better understand this finding, it is important to place it into an evolutionary framework, and to consider the adaptive function of reactions to specific emotional expressions (Schmidt and Cohn, 2001; Öhman et al., 2001). Both angry and fearful expression may be perceived as potential threatening information, but they emerge for different reasons, and therefore require different responses. Fearful expressions are shown in response to a threat in the environment, and the individual perceiving this expression on someone’s face needs to detect the source of the threat, and is therefore orienting attention to this face (Öhman, 2008). The white sclera of the human eye (Kobayashi and Kohshima, 1997) attracts this unconscious orienting response, mediated by the amygdala, from an early age on (Whalen et al., 2004; Jessen and Grossmann, 2014; for a review see Emery, 2000). Angry expressions, on the other hand, may represent a direct threat from a social partner and may therefore result in avoiding the other’s gaze (Marsh et al., 2005). In support of these predictions, we found that participants looked longer at fearful, but shorter on angry faces, regardless of their cultural background.

Other approaches, which categorize emotions based on the different reaction tendencies they elicit, come to a different conclusion. Williams et al. (2005) propose that not fearful, but angry faces signal an immediate threat to recipients and should therefore attract their attention, while fearful faces divert recipients’ attention, as the potential threat comes from a source in the environment, but not from the signaler itself. Similarly, Adams and Kleck (2005), who studied the impact of gaze direction of emotional expressions on approach and avoidance responses, described anger as an approach-oriented emotion, as direct gaze increased the perceived intensity of angry faces, but fear as avoidance-oriented emotion, since averted gaze increased the perceived intensity of fearful expressions. These predictions resulting from different reaction tendencies – longer looks toward angry than fearful facial expressions – are not supported by our findings. However, unlike Adams and Kleck (2005), we did not manipulate gaze direction, but only used direct gaze. Furthermore, since our stimuli depicted adults, who could be perceived as physically or mentally superior by our participants, we cannot exclude an impact on children’s and adolescents’ gaze patterns. Thus, an alternative explanation for our findings of shorter fixation durations on angry faces is that they perceived anger in adult faces as more threatening than adult participant would do. To address this issue, future studies should use stimuli resembling young participants’ ages, and should vary gaze direction across angry and fearful faces (see van Rooijen et al., 2017).

A possible explanation for these inconsistent findings across studies regarding the attentional bias to either fearful or angry faces comes from Mogg et al. (2007). They differentiated between reflexive and reflective attention, and suggest that initially, both angry and fearful faces automatically attract attention. At a later stage, attention is averted from angry faces, while for fearful faces, attention is maintained to identify the most appropriate response. This suggests that different mechanisms may underlie the processing of negative faces, depending on how long such stimuli are presented. Indeed, several studies with children and adolescents between 3 and 18 years draw different conclusions with regards to attentional biases toward anger or fear, depending on whether early fixations (<120 ms after stimulus onset) or maintained attention (dwell time across stimulus presentation) are measured (Pool et al., 2016; Lisk et al., 2019). An attentional bias toward angry faces is evident during initial fixations (Shechner et al., 2013), while maintained gazes at angry faces are avoided (Lisk et al., 2019). Thus, unlike initial orienting, maintained attention – as measured in our study – suggests some volitional “top-down control,” which is expected to increase with children’s age (Lagattuta and Kramer, 2017). In our study, age had no effect on the overall fixation duration of different types of emotions, but it is important to keep in mind that unlike in these studies, our sample did not include preschool children.

For happy faces, only Germans showed a bias for happy over angry or neutral faces. A possible explanation for this variability between groups is that positive emotions elicit more flexible reactions, since they broaden the recipient’ attention and facilitate social interactions (Fredrickson, 1998), but do not require an immediate and appropriate response like negative emotions. However, as only happy faces have been presented, it remains unclear if this finding can be generalized to other positive emotions (Sauter, 2010). Clear evidence for a positivity bias was found in studies using a greater variety of positive information (e.g., erotic pictures, food, babies; Pool et al., 2016).

Together, our findings suggest a culture-independent bias toward fearful, but not angry facial expressions, while there is more variability between cultural groups with regard to their responses to positive faces. This may suggest that the different, emotion-specific responses to negative facial expressions are adaptive and most likely shared across humans, since unlike positive emotions, fearful and angry faces require very specific, but different responses, to avoid potential harmful consequences.

Although this conclusion is tempting, it is important to consider some limitations of this study. First, based on the comparison of small samples from only two cultural groups, it is impossible to conclude that our finding of an attentional bias toward fearful, but not angry faces represents a universal pattern. Second, unlike Germans, the ≠Akhoe Hai||om did not look at facial expressions from their own ethnical group, which could have influenced their emotion recognition (Elfenbein and Ambady, 2002). Therefore, we cannot rule out the possibility that differences between cultural groups regarding happy faces are caused by the varying familiarity with the stimuli. This, however, seems not very likely given the similarities between groups with regard to their processing of negative emotions. Still, we have to consider the possibility that the ≠Akhoe Hai||om interpret facial emotions differently compared to Germans, as the universality hypothesis of basic emotion perception has been repeatedly questioned (Jack et al., 2012; Gendron et al., 2014), and since contextual information seems crucial for interpreting facial expressions (Crivelli et al., 2016). Third, only faces of adults, but not peers, were presented, which might have influenced the young participants’ fixations on anger. However, it is important to note that this is not a limitation unique to our study, as most research with children and adolescents is not using age-matched facial expressions. Fourth, since our sample only included adolescents and children, it remains unclear if our findings can be generalized to adults, as adolescents’ face processing capabilities may not fully resemble those of adults yet (Batty and Taylor, 2006), and since their bias toward negative stimuli develops throughout adolescence (Yurgelun-Todd and Killgore, 2006; Thomas et al., 2007). Despite these shortcomings, this study is a first step toward a better understanding of the cross-cultural similarities and differences in human emotion perception and provides evidence for the special relevance of fearful faces compared to facial expressions of other emotions.

## Data Availability Statement

All datasets generated for this study are included in the article/Supplementary Material.

## Ethics Statement

Ethical approval was not provided for this study on human participants because it did not involve any invasive techniques, ethically problematic procedures, or deception. Therefore, according to the regulations on freedom of research in the German Constitution [§5 (3)], it did not require approval by an Institutional Review Board. However, since our sample included participants from an indigenous group in Namibia, we obtained approval by the “Working Group of Indigenous Minorities in Southern Africa,” located in Windhoek, Namibia. In Germany, parents’ informed consent was obtained in the written form. In Namibia, however, this was problematic, since most parents were not able to read or write. Therefore, since this study was conducted at the school, we obtained permission by the principal of this school and obtained the participant’s additional consent either verbally (“yes”) or visually (“nodding”).

## Author Contributions

CM and CP were responsible for designing this study and for collecting and analyzing the data. CM also contributed to writing the manuscript. IW and ST were involved in designing this study and contributed to drafting this manuscript. KL contributed to planning the study, collected parts of the data and was involved in writing the manuscript.

## Conflict of Interest

The authors declare that the research was conducted in the absence of any commercial or financial relationships that could be construed as a potential conflict of interest.

## Footnotes

1 https://faces.mpdl.mpg.de
